# PKR activity modulation by phosphomimetic mutations of serine residues located three aminoacids upstream of double-stranded RNA binding motifs

**DOI:** 10.1038/s41598-021-88610-z

**Published:** 2021-04-28

**Authors:** Teresa Cesaro, Yohei Hayashi, Fabian Borghese, Didier Vertommen, Fanny Wavreil, Thomas Michiels

**Affiliations:** 1 de Duve Institute, Université Catholique de Louvain, VIRO B1.74.07, 74, Avenue Hippocrate, 1200 Brussels, Belgium; 2 PHOS Unit and MASSPROT Platform, de Duve Institute, Université Catholique de Louvain, Brussels, Belgium; 3 Present Address: Frontier Sciences Unit, Department of Medical Innovations, Otsuka Pharmaceutical Co., Ltd., Tokushima, Japan

**Keywords:** Viral host response, Stress signalling, Innate immunity

## Abstract

Eukaryotic translation initiation factor 2 alpha kinase 2 (EIF2AK2), better known as PKR, plays a key role in the response to viral infections and cellular homeostasis by regulating mRNA translation. Upon binding dsRNA, PKR is activated through homodimerization and subsequent autophosphorylation on residues Thr446 and Thr451. In this study, we identified a novel PKR phosphorylation site, Ser6, located 3 amino acids upstream of the first double-stranded RNA binding motif (DRBM1). Another Ser residue occurs in PKR at position 97, the very same position relative to the DRBM2. Ser or Thr residues also occur 3 amino acids upstream DRBMs of other proteins such as ADAR1 or DICER. Phosphoinhibiting mutations (Ser-to-Ala) introduced at Ser6 and Ser97 spontaneously activated PKR. In contrast, phosphomimetic mutations (Ser-to-Asp) inhibited PKR activation following either poly (I:C) transfection or virus infection. These mutations moderately affected dsRNA binding or dimerization, suggesting a model where negative charges occurring at position 6 and 97 tighten the interaction of DRBMs with the kinase domain, thus keeping PKR in an inactive closed conformation even in the presence of dsRNA. This study provides new insights on PKR regulation mechanisms and identifies Ser6 and Ser97 as potential targets to modulate PKR activity for therapeutic purposes.

## Introduction

Four protein kinases were shown to phosphorylate the eucaryotic translation initiation factor 2α (eIF2α) in response to diverse stress stimuli^1^. They were thus named eukaryotic translation initiation factor 2 alpha kinase-1, -2, -3, and -4, but are usually referred to as HRI, PKR, PERK and GCN2, respectively.

PKR, is a serine-threonine and tyrosine kinase, whose expression is enhanced by type-I interferon (IFN) and whose activation follows binding to double-stranded (ds) RNA. eIF2α phosphorylation leads to blockade of preinitiation complex formation at the initiation codon and hence to translation blockade and consequent stress granule formation^2^. PKR is known to play a key role in the antiviral defence: it senses dsRNA molecules generated during replication of DNA and RNA viruses, and gives rise to a potent antiviral response by blocking viral mRNA translation and ultimately leading to infected cell apoptosis^3^.

PKR is a 551 amino acid-long protein that contains two dsRNA binding motifs (DRBMs) at its N-terminus, and a kinase domain at its C-terminus. The catalytic domain is composed by an N-lobe that contributes to dimerization and a C-lobe containing the substrate-binding pocket^4,5^.

In its inactive state, PKR stays in a so-called closed conformation, in which the second DRBM^6^ is tightly interacting with the kinase domain, hiding the kinase substrate-binding pocket and thereby preventing its random activation^7^.

In the course of viral infection, dsRNA molecules are formed and are sensed by the DBRMs of the kinase. Although both DRBMs are involved in dsRNA binding, the RNA-binding site in DRBM2 appears to be less conserved with known RNA-binding sites of other proteins than that in DRBM1. Many studies have speculated about the importance of the DRBM1 for binding to dsRNA^8^. This interaction is nucleic acid-specific but not sequence-specific. Bevilacqua et al. showed that the DRBMs of PKR bind exclusively dsRNA and not dsDNA or RNA–DNA duplexes. They speculated about the possibility of contact mediated by hydrogen bonding between the hydrophilic residues in the RNA-binding site of PKR and the 2′-OHs of the sugars^9^. In addition to this interaction, one ion pair has been found between the negatively charged phosphates of the nucleic acid and the positively charged residues on the DRBMs of PKR. Mutagenesis analysis identified one lysine (K60) and one leucine (L75) in the first DRBM1, as being critical for dsRNA binding^10^. When either residue is mutated to alanine, binding to dsRNA is completely abrogated. Other lysines like K64, K69, K150 and K154 have been described as important to various degrees but not necessary to mediate PKR binding to dsRNA^10^. Binding to dsRNA molecules would trigger a conformational change, switching PKR from a closed to an open conformation^11^. Binding to dsRNA is necessary for promoting the successive steps in the activation process of the protein: mutations that disrupt dsRNA binding interfere with dimerization and autophosphorylation^12,13^. Many studies explored how dsRNA binding is promoting dimerization and autophosphorylation of PKR. One dsRNA molecule likely brings together two PKR molecules, thus stabilizing a PKR dimer^11^. Next, the kinase pocket of the protein, released from DRBM2, would autophosphorylate PKR at different serines and threonines, among which the best known are the residues Thr446 and Thr451, markers of the kinase activation^14^. Different models have been proposed to explain the process of autophosphorylation: cis autophosphorylation (one protomer within a dimer phosphorylates on its own), inter-dimer phosphorylation (cross-phosphorylation between different dimers) or intra-dimer phosphorylation (one protomer in a dimer phosphorylates the activation loop of its partner). The most accredited one remains the cis-intra dimers phosphorylation model^15^. Autophosphorylation of PKR at Thr446 and Thr451 is required for the specific recognition of its substrate eIF2α^12^.

In addition to dsRNA, cellular proteins can regulate PKR activation, in either a positive or a negative fashion. PACT (protein activator of interferon induced protein kinase EIF2AK2) and Rax, the mouse homolog, are PKR activators. They can activate PKR in vitro in the absence of dsRNA through direct protein–protein interaction^16,17^. PACT interacts with PKR in response to a variety of cellular stresses: it has been reported to activate PKR in vitro as well in vivo, after an oxidative stress^18^. PACT was also shown to be required for PKR activation after vesicular stomatitis virus infection^19^. TRBP (TAR RNA binding protein 2) and P58IPK (alias DNAJC3, heat shock protein family (Hsp40) member C3) are two other proteins that regulate PKR through protein–protein interaction. In contrast to PACT, they act as PKR inhibitors^20,21^.

Once activated, PKR phosphorylates the translation initiator factor eIF2α at Ser51. Upon phosphorylation of eIF2α, mRNA translation is inhibited. As a result, large complexes of 48S mRNPs and associated proteins accumulate in cytoplasmic aggregates, called stress granules^2^. Canonical stress granules markers include TIA-1, G3BP1 and several other proteins, which help the nucleation of these aggregates^20^.

Viruses have developed a number of mechanisms to antagonize PKR, acting upstream or downstream its activation step. They do so by using viral products or by hijacking cellular proteins^3^. As an example, in the *Picornaviridae* family, we have previously shown that L protein of Theiler’s murine encephalomyelitis virus (TMEV, *Cardiovirus* genus) can inhibit PKR activation by preventing dsRNA recognition by PKR^22^.

Understanding the regulation of PKR is not only key to understanding of antiviral responses and escape mechanisms but also constitutes a way to envision therapeutic approaches aimed at dampening PKR overactivation occurring in a number of autoimmune diseases such as Aicardi-Goutières syndrome or rheumatoid arthritis^23,24^.

In the latter case, Wang et al. showed, in a rat model, that, treatment with the PKR inhibitor C16 significantly improved the course of the disease. These authors observed that PKR contributed to the release of HMGB1, which causes inflammation when released in the synovial fluid^25^.

In this work, we examined the possibility of PKR activity control by phosphorylation. This work stems from a fortuitous observation that unexplored PKR phosphorylation sites turned out to be phosphorylated in cells infected with TMEV.

## Results

### Serine 6 can be phosphorylated in virus-infected cells

**Figure 1:**
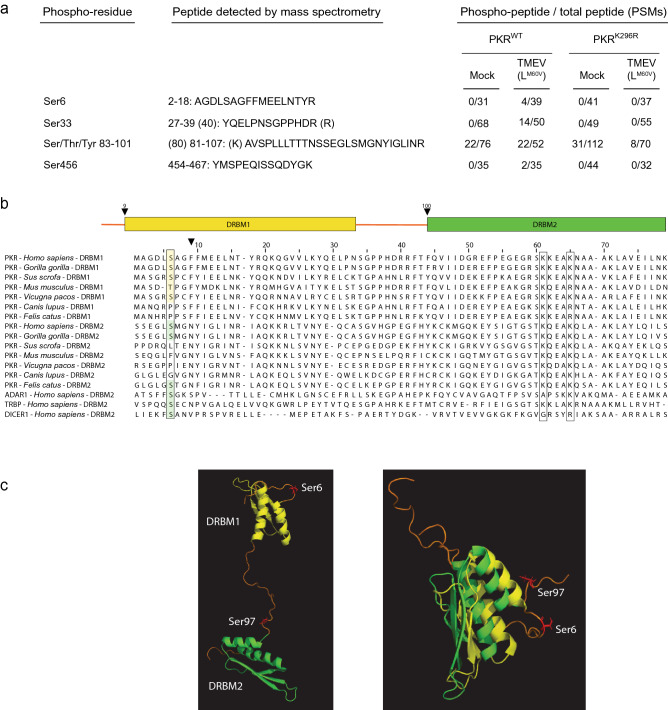
PKR serine 6 can be phosphorylated in TMEV-infected cells. (**a**) Phosphorylation sites identified after PKR immunoprecipitation from HeLa (PKR^WT^) or from PKR-KO cells transduced to express a kinase-dead PKR mutant (PKR^K296R^), thus deficient for autophosphorylation. Phosphorylated PKR peptides were identified in mock-infected cells and in cells infected with the L^M60V^ TMEV mutant. The PSMs (peptide spectrum match) ratio of phosphorylated on total corresponding peptides are shown for the detected phospho-peptides. (**b**) Amino acid sequence alignment of the first and second dsRNA binding motifs (DRBMs—yellow and green frames) from the indicated species. (**c**) Conserved position of Ser6 and Ser97 relative to DRBMs. The DRBM1 and 2 are shown in yellow and green, respectively. Ser6 and Ser97 are shown in red and labeled in the unstructured regions preceding the DRBMs. On the right a merged picture was generated by superimposing the two DRBMs and the surrounding regions (NMR structure of PKR^8^, PDB code 1QU6 ). Images were generated using the PyMOL molecular Graphic system, Version 2.1, Schrödinger, LLC.

In order to map PKR phosphorylation sites that may contribute to PKR regulation, PKR was immunoprecipitated from HeLa cells that were either mock-infected or infected with a TMEV strain expressing a mutant L protein (L^M60^^V^) that fails to block PKR activation^22^. Immunoprecipitated samples, collected after 10 h of infection, were resolved by SDS-PAGE and Coomassie blue-stained bands around 70Kda (expected molecular weight for PKR) were analyzed by mass spectrometry (Fig. 1a). Besides known PKR phosphorylation sites such as Ser33, our attention was caught by a yet undescribed phosphorylation site occurring at the level of serine 6 that is located three amino acids before the first DRBM (Fig. 1b). Since then, phosphorylation of this residue has been confirmed in at least one phosphoproteomic report^26^. A Ser residue is conserved at the same position in the PKR sequence of other primates such as *Gorilla gorilla*. *Mus musculus* PKR contains a threonine equivalent to human S6 upstream of DRBM1. Interestingly, although it was not documented to be phosphorylated, a serine (S97) is located, in PKR, at the very same position relative to the second DRBM (Fig. 1c). This serine residue is conserved in many species including *Danio rerio.* A phosphorylatable residue is also found at the same position (− 3) relative to the dsRBD in other human proteins such ADAR1 (DRBM2), TRBP (DRBM2), and DICER.

To test whether Ser6 phosphorylation was mediated by PKR itself or by another cellular kinase, a similar mass spectrometry analysis was performed after PKR immunoprecipitation from PKR-KO HeLa cells transduced with the lentiviral vector TC27 that expresses a kinase-dead PKR mutant (K296R)^27^ (Fig. 1a). In contrast to some unidentified residues contained in the Ser/Thr-rich peptide 83–101, Ser6 was not phosphorylated in cells expressing kinase-dead PKR, suggesting that Ser6 is an autophosphorylation site (Fig. 1a).

### Mutations of Ser6 and Ser97 regulate PKR activation

**Figure 2:**
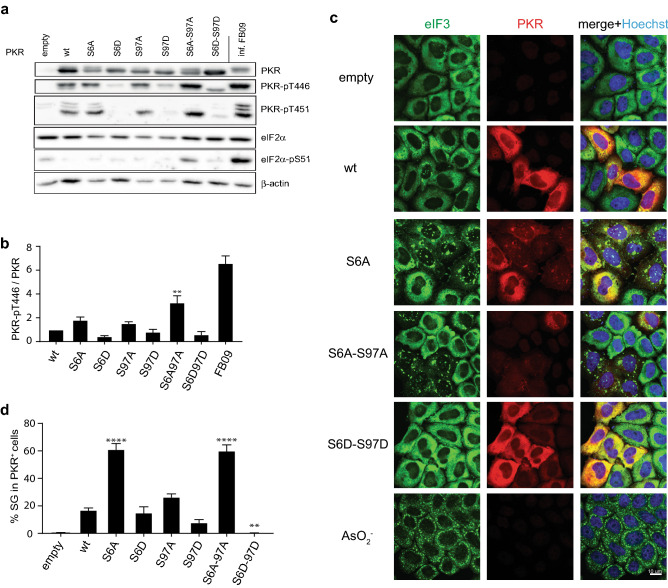
Mutations of Ser6 and Ser97 regulate PKR activation. PKR-KO cells were transfected with vectors expressing either wild type (wt) or mutant PKR, as indicated. After 48 h, cells were lysed for western blot or fixed for immunofluorescence analysis. (**a**) Representative western blots showing total and phosphorylated forms of PKR and eIF2α. β-actin was detected as a loading control. (**b**) Quantification of the phospho-Thr446 (PKR-pT446) / total PKR ratio on western blots (n = 4). (**c**) Confocal microscopy images showing spontaneous stress granule formation (eIF3, green) in cells transfected with PKR (red) expressing vectors. Arsenite treatment (NaAsO_2_) for 30′ at 37 °C was used as a positive control for SG formation. (**d**) Percentage of stress granule-positive cells counted among PKR-positive (PKR^+^) cells. Western blots were quantified by chemiluminescence using a CCD camera (Fusion Solo-S, Vilber) and quantified within the limits of the dynamic range, using software Bio1D version 15.08. Graphs (generated with Microsoft Excel 2011) show mean ± SEM (n = 4); 1-way ANOVA (Graphpad Prism 7) was used to compare PKR mutants to wt PKR. Microscopy images were acquired using Zen (Zeiss).

The human PKR sequence was mutated at position 6 or 97 to either alanine, to prevent phosphorylation or to aspartate, a residue mimicking a phosphorylated serine. PKR constructs were also obtained with both Ser residues mutated to Ala or Asp. To test the impact of the mutations on PKR activation, PKR-KO HeLa cells were transfected with the wildtype and mutant PKR expressing vectors. Spontaneous PKR activation, as assessed by Thr446 phosphorylation, was analyzed by western blot 48 h after transfection. We observed that PKR-S6A, -S97A and -S6A-S97A were spontaneously activated more strongly than the wt control (Fig. 2a-b). Thr451 phosphorylation followed a similar pattern (Fig. 2a). eIF2α phosphorylation was readily detectable after expression of PKR-S6A-S97A, confirming that the double Ala mutation increased PKR kinase activity. Spontaneously activated PKR mutants tended to be less expressed, likely as a consequence of to their self-inhibitory role on translation.

In contrast, Asp mutants tended to exhibit decreased phosphorylation as compared to the wt control (Fig. 2a,b, Fig S1). We also examined the impact of the phosphoinhibitory (Ala) and phosphomimetic (Asp) PKR mutations on stress granule formation in transfected cells, which, in our hands, turned out to be a robust readout for PKR activity. In agreement with the above PKR activation data, confocal microscopy analysis (Fig. 2c,d) showed increased stress granule formation in cells transfected with the PKR-Ala mutants and decreased stress granule formation in cells transfected with the PKR-Asp mutants.

Taken together, our data show that phosphoinhibitory Ser-to-Ala mutations of Ser6 and Ser97 increase spontaneous PKR activity while phosphomimetic Ser-to-Asp mutations decrease PKR activity.

### PACT is not required for spontaneous activation of Ser6- and Ser97-to-Ala PKR mutants

**Figure 3:**
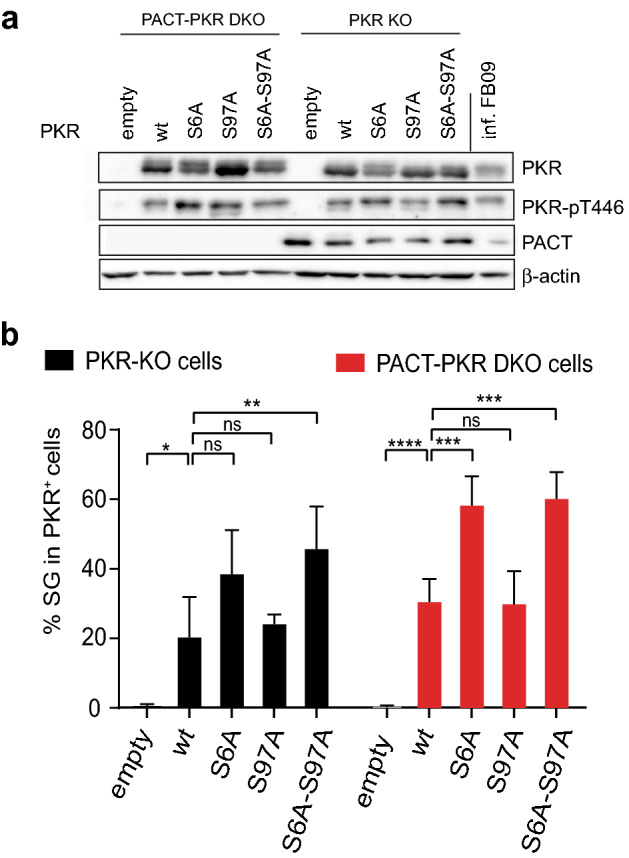
PACT is not required for spontaneous activation of Ser6- and Ser97-Ala PKR mutants. PACT-PKR DKO cells and PKR-KO cells were transfected with plasmids expressing either wt PKR or indicated PKR mutants. After 48 h, PKR activation (phospho-Thr446) was analyzed by western blot (**a**) and stress granule formation was quantified in transfected cells using eIF3 detection by immunofluorescence (**b**). (**a**) Detection of phospho-Thr446 PKR (PKR-pT446), PKR and PACT by western blot. Note the similar activation of PKR in PACT-positive and PACT-deficient cells. β-actin was detected as a loading control. Acquisition was made with a CCD camera (Fusion Solo-S, Vilber) using the Bio1D software (version 15.08). (**b**) Graph (generated with Microsoft Excell 2011) showing the percentage of stress granule-positive cells counted among PKR^+^ cells, for PACT-PKR DKO and PKR-KO cell lines (mean ± SEM) (n = 4). 2-way ANOVA (Graphpad Prism 7) was used to compare mutant and wt PKR in each cell line.

PACT is a protein that was reported to contribute to PKR activation through direct protein–protein interaction^17,28^. To test whether PACT was responsible for PKR activation mediated by S6A, S97A and S6A-S97A, we compared spontaneous PKR activation levels in PACT-PKR double KO and PKR-KO HeLa cells where wt or mutant PKR was re-expressed. Phospho-PKR detection by western blot and SG counts (Fig. 3, Fig S2) in cells transfected with PKR expression vectors showed similar PKR activation patterns in PACT-KO and PACT-sufficient cells. These results suggest that spontaneous PKR activation observed for S6A, S97A and S6A-S97A mutants is not dependent on PACT.

### DRBMs are needed for full activation of PKR-S6A-S97A

**Figure 4:**
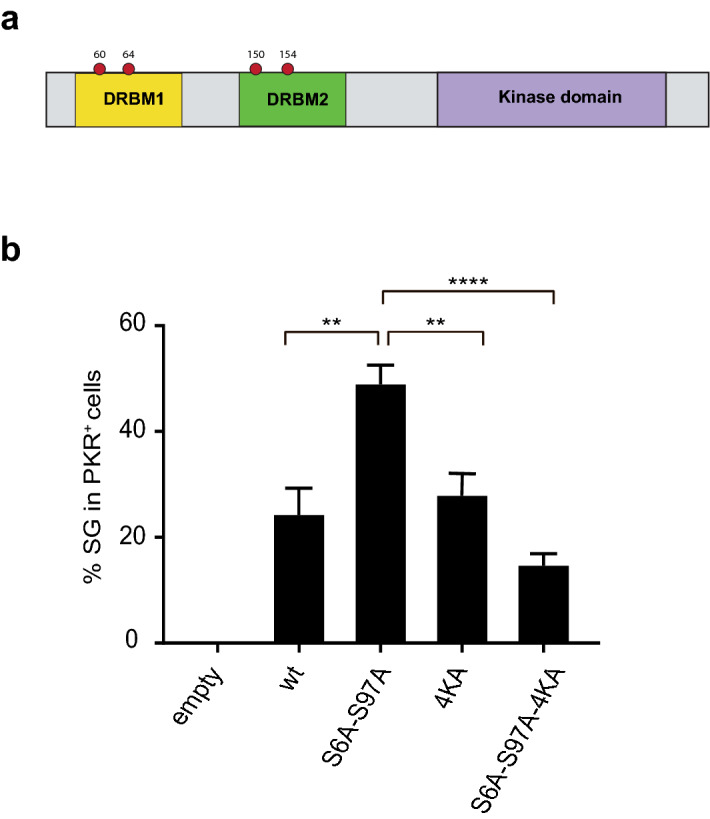
dsRNA binding contributes to spontaneous activation of S6A-S97A PKR mutant. (**a**) Schematic representation of the main PKR domains. The coordinates on the top are those of the four lysines mutated to alanines in order to generate a DRBM-dead PKR (PKR-4KA). (**b**) PKR-KO cells were transfected with plasmids expressing PKR mutants and fixed 48 h post-transfection for eIF3 and PKR detection by immunofluorescence. Graphs (generated with Microsoft Excel 2011) show the mean and SEM of the percentages of stress granule-positive cells counted among PKR^+^ cells (n = 4). 1-way ANOVA (Graphpad Prism 7) compared each PKR condition to the S6A-S97A PKR mutant condition.

The above data suggest that phosphoinhibitory mutations S6A and S97A favour a conformational change similar to the one occurring after dsRNA binding, i.e. the “opening” of the kinase due to the dissociation of the kinase domain from the dsRNA binding domains. To test if the spontaneous activation of PKR-S6A, -S97A and -S6A-S97A was still dependent on binding to dsRNA, we generated a DRBM-dead PKR mutant by replacing lysine residues K60, K64, K150 and K154 involved in dsRNA binding, by alanine residues (PKR-4KA) (Fig. 4a). We also combined these mutations together with the Ser-to-Ala mutations at position 6 and 97 (PKR-S6A-S97A-4KA). HeLa PKR-KO cells were transfected with PKR coding vectors for 48 h and stress granules were detected by immunofluorescence as a readout of PKR activity (Fig. 4b). On the one hand, stress granules were still formed upon transfection of the DRBM-dead 4KA mutant construct but not upon transfection of the empty vector, suggesting that PKR, when overexpressed by transfection, can be activated without binding dsRNA. On the other hand, the percentage of stress granule-positive cells was significantly lower for cells expressing PKR-S6A-S97A-4KA than for cells expressing the PKR-S6A-S97A mutant that conserved dsRNA binding ability, suggesting that dsRNA binding was still an activation stimulus for the “overactivated” PKR-S6A-S97A mutant.

### The double S6D-S97D mutation prevents PKR activation by dsRNA

**Figure 5:**
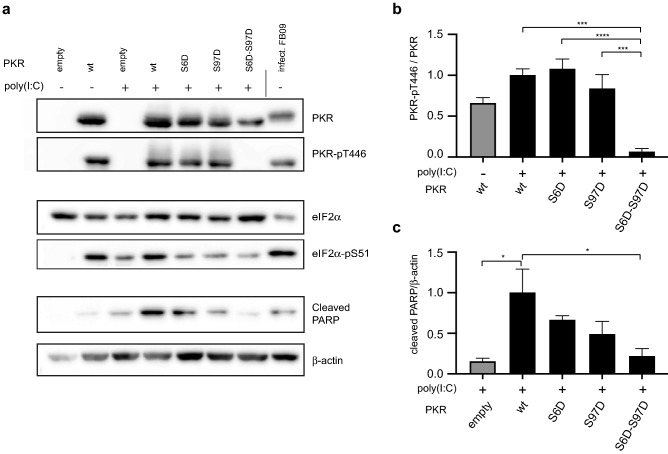
Combination of S6D and S97D phosphomimetic mutations prevents PKR activation by poly(I:C). PKR-KO cells were transfected either with wt PKR or Ser6- and/or Ser97-to-Asp PKR mutants for 40 h. Cells were next transfected with poly(I:C) for 8 h and then lysed for western blot analysis. The FB09 sample (HeLa cells infected with L^M60V^-mutant TMEV for 12 h) was used as positive control for PKR activation (**a**) Western blot detection of PKR, PKR-pT446, eIF2α, eIF2α-pS51, cleaved PARP and β-actin. (**b**) Quantification of the PKR-pT446 / PKR ratio. (mean ± SEM, n = 4). (**c**) Quantification of apoptosis by the cleaved PARP / β-actin ratio. (mean ± SEM, n = 4). 1-way ANOVA was used for multiple comparisons between poly(I:C)-treated samples. Western blots were quantified by chemiluminescence using a CCD camera (Fusion Solo-S, Vilber) and quantified within the limits of the dynamic range, using software Bio1D version 15.08. Graphs were generated with Excel 2011 (Microsoft). Statistical analysis was performed using Prism7 (Graphpad).

PKR-S6D, -S97D and -S6D-S97D mutants showed decreased spontaneous activation as compared with wild type PKR (Fig. 2). We investigated the ability of these mutants to be activated by poly(I:C) transfection. Poly(I:C) was thus transfected into the cells expressing PKR-S6D, -S97D and -S6D-S97D and PKR Thr446 phosphorylation was followed by western blot. As shown on Fig. 5 (full-size blots available as Fig. S3), a single mutation of either serine 6 or 97 to aspartate did not prevent PKR activation by poly(I:C). In contrast, the double S6D-S97D mutation dramatically inhibited PKR activation. PKR activation by poly(I:C) leads to cell death by apoptosis^29^. PARP cleavage, taken as a readout for apoptosis, fairly correlated with PKR Thr446 phosphorylation and was minimal for the S6D-S97D mutant (Fig. 5c).

**Figure 6:**
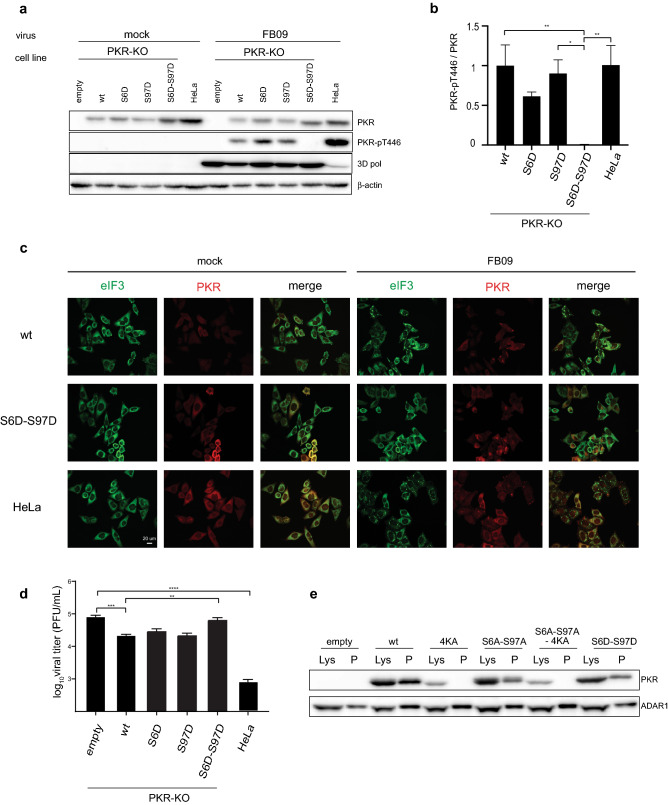
Combination of S6D and S97D phosphomimetic mutations prevents PKR activation by viral infection and enhances virus production. Control HeLa cells and PKR-KO cells transduced to re-express indicated PKR constructs were either mock-infected or infected with the L^M60V^ TMEV mutant (FB09). (**a**) Western blot showing the detection, 12 h post-infection, of PKR, phospho-Thr446 PKR (PKR-pT446), 3D polymerase as an infection control and β-actin as a loading control. (**b**) Graph showing PKR activation (PKR-pT446 / PKR ratio) as quantified from the western blots (mean ± SEM, n = 6). 1-way ANOVA was used for multiple comparisons. (**c**) Confocal microscopy images showing the co-immunostaining of PKR (red) and eIF3 (green) in cells that were mock- or virus-infected for 8 h. (**d**) Quantification, by plaque assay, of FB09 (L^M60V^ mutant TMEV) production 12 h after infection of control HeLa cells and PKR-KO cells expressing indicated PKR variants (mean ± SEM, n = 4). 1-way ANOVA was used for multiple comparisons. (**e**) Binding of PKR variants to poly(I:C). PKR-KO cells transfected for 48 h with plasmids expressing wt or mutant PKR. Cell lysates were collected, incubated with biotinylated poly (I:C), and pulled down with streptavidin beads. Western blots are presented showing PKR detection in cell lysates (Lys) and in pulled down fractions (P). ADAR1 was detected as a dsRNA binding protein control. Microscopy images were acquired using Zen (Zeiss). Western blots were quantified by chemiluminescence using a CCD camera (Fusion Solo-S, Vilber) and quantified within the limits of the dynamic range, using software Bio1D version 15.08. Graphs were generated with Excel 2011 (Microsoft). Statistical analysis was performed using Prism7 (Graphpad).

We next questioned the possibility to activate PKR-S6D, -S97D and -S6D-S97D by viral infection, the biological PKR agonist. Therefore, PKR-KO HeLa cells, transduced with lentiviral vectors to stably express wild type or Ser-to-Asp PKR mutants, were infected for 12 h with FB09, a L^M60^^V^ TMEV mutant that lost PKR antagonism^22^. As in the case of poly(I:C) transfection, western blot analysis (Fig. 6a,b, Fig S4) showed no activation of the double Asp PKR mutant S6D-S97D after viral infection. Accordingly, PKR-S6D-S97D expression failed to trigger stress granule formation upon FB09 infection (Fig. 6c). Virus yield, as measured by plaque assay, was not inhibited by re-expression of PKR-S6D-S97D in PKR-KO cells, in contrast to wild type PKR re-expression (Fig. 6d).

As the double Ser6- Ser97-to-Asp PKR mutant was activated neither by poly(I:C) nor by viral infection, we hypothesized that phosphate-mimicking negative charges brought just upstream the DRBMs could act as a repellent for the negatively charged dsRNA and thus prevent PKR binding to dsRNA. We thus examined dsRNA binding ability of PKR-S6D-S97D using a biotinylated poly(I:C) pull down assay. As shown on Fig. 6e (full-size blots available on Fig. S5), the double phosphomimetic (S6D-S97D), as the double phospho-inhibiting (S6A-S97A) mutation reduced but did not block PKR binding to dsRNA, contrary to the DRBM-dead (4KA) mutation that was used as a control.

### Dimerization of PKR mutants

**Figure 7:**
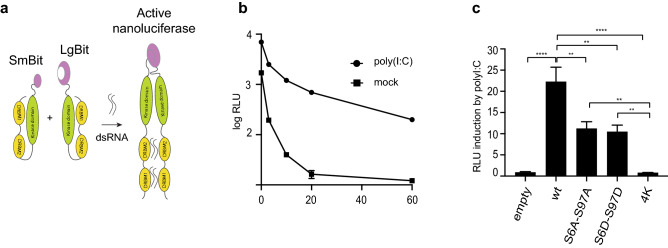
PKR dimerization assay. (**a**) Principle of the PKR dimerization assay based on split nanoluciferase: PKR constructs C-terminally tagged with nanoluciferase SmBit and LgBit fragments are co-expressed in PKR-KO cells. Upon poly(I:C) transfection, PKR dimerization leads to the assembly of active nanoluciferase. (**b**) Kinetics of nanoluciferase activity measured after substrate addition, for wt PKR, in mock-transfected cells and in cells transfected with poly(I:C) for 2 h before substrate addition. (**c**) Graphs show the mean and SEM of nanoluciferase activity induction following poly(I:C) transfection in cells that co-expressed wt or mutant PKR linked to the Sm and Lg Bits of nanoluciferase (homodimerization assay). Luciferase activity was measured 5 min after substrate addition. Significance is shown for multiple one-way ANOVA comparisons with WT PKR and with the negative control 4 K. Graphs were generated with Excel 2011 (Microsoft). Statistical analysis was performed using Prism7 (Graphpad).

The above results suggest that Ser-to-Ala mutations of Ser6 and Ser97 facilitate the conformational change that leads to PKR dimerization and activation. The split nanoluciferase reporter system^30^ was used in an attempt to examine the dimerization ability of PKR mutants (Fig. 7a). Expression vectors were constructed that express wt PKR or PKR mutants C-terminally fused to the large (LgBit) and small (SmBit) parts of the nanoluciferase. PKR-KO cells were transfected with the corresponding LgBit and SmBit PKR constructs and subsequently transfected with poly(I:C). Nanoluciferase activity was then monitored as a surrogate marker for PKR dimerization. Data presented in Fig. 7b,c surprisingly suggest that Ser-to-Ala mutations slightly decreased dimerization despite the PKR-activating influence of these mutations. Ser-to-Asp mutations inhibited dimerization to the same extent as the Ser-to-Ala mutations. This slight dimerization inhibition of the latter mutant, however, unlikely explains the strong inhibition of PKR activation observed above (Figs. 5, 6).

## Discussion

PKR is a critical protein kinase located at the crossroad of pathogen-associated molecular pattern sensing and interferon effector pathways. It has a potent, broad range, antiviral activity. Knocking down or knocking out PKR dramatically increases susceptibility of cells or mice to viral infection^31,32^. As a witness of PKR antiviral potency, numerous viruses evolved mechanisms to antagonize PKR activity (reviewed in^3^).

On the other hand, aberrant PKR activation may be detrimental to the host as PKR activity typically leads to mRNA translation blockade and ultimately to cell apoptosis. Abnormal PKR activation was documented in several autoimmune diseases such as rheumatoid arthritis, lupus erythematosus or interferonopathies such as the Aicardi-Goutières syndrome. Aicardi-Goutières syndrome can be linked to loss-of-function mutations in *ADAR1*, the gene encoding the double-stranded RNA-specific adenosine deaminase-1^33^. In the latter case, excess of endogenous dsRNA resulting from the absence of dsRNA destabilizing activity of ADAR1 may not only trigger the MDA5 sensor leading to interferon production, which upregulates PKR gene transcription, but also contribute to PKR activation by dsRNA binding^34^. Aberrant PKR activity has also been associated with neurodegenerative disorders and cancer^35,36^. In the latter case, both cancer promoting and cancer inhibiting properties have been imparted to PKR^36^. PKR activity obviously requires fine tuning.

**Figure 8:**
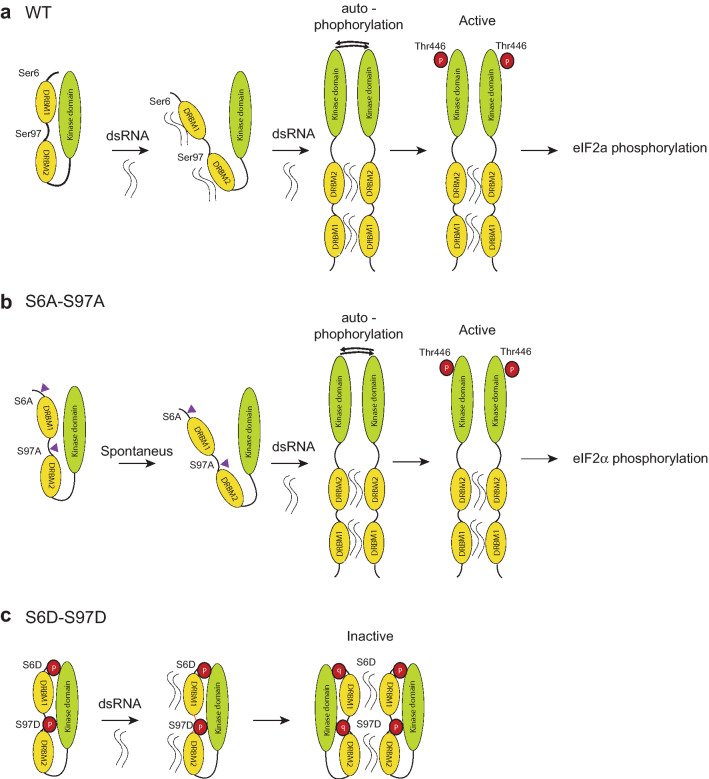
Model: Ser6 and Ser97 mutations impact the stability of the closed PKR conformation. (**a**) Wild type PKR: dsRNA binding by DRBM1 and/or DRBM2 destabilizes the interaction between the RNA binding domain and the kinase domain, leading to opening, dimerization, autophosphorylation and activation of the kinase activity. (**b**) The Ser-to-Ala mutations spontaneously destabilize the close conformation and facilitate opening of PKR upon dsRNA binding. (**c**) Ser6 and 97 phosphorylation or Ser-to-Asp phosphomimetic mutations prevent neither dsRNA binding nor dimerization but likely contribute to maintain PKR in a close, inactive conformation.

PKR regulation occurs at multiple levels. Gene transcription can be upregulated by interferon. Catalytic activity of the kinase depends on a cascade of events that includes dsRNA binding, dimerization and autophosphorylation (Fig. 8a). These steps can further be modulated by interaction of PKR with proteins such as PACT^17^, TRBP^21^ and p58IPK^20^ by RNA–protein interactions involving helicases^37^, and by post-translational modifications such as SUMOylation^38^. More recently, PKR was reported to be regulated through binding of a non-coding RNA nc886, which can act either as a strong PKR repressor or as a weak positive regulator, according to its conformation^39,40^.

PKR phosphorylation has been well-documented. Phosphorylated residues have been mapped in the catalytic domain (Thr446, Thr451) but also in the central region between the two DRBMs (Ser242, Thr255, Thr258). Autophosphorylation of Thr446 and Thr451 is a prerequisite for target (eIF2α) phosphorylation by PKR. Additional phosphorylation sites were identified, including Ser83 and Thr88, 89 and 90, located in the spacer region of the DRBMs^41^. Phosphorylation of these residues contributes to PKR activation.

Wang et al.^42^ showed that PKR could use autophosphorylation to exert a negative feedback on its own activity. They found that phosphorylation of Ser33 and Thr42 inhibits dimerization and thus terminates PKR-dependent control of protein translation. Additional PKR phosphorylation sites were identified in phosphoproteomic screens (https://www.phosphosite.org/homeAction) but were not studied in detail.

We observed PKR Ser6 phosphorylation. This residue is located three amino acids upstream of the first DRBM, in the very same relative position as serine 97, which is located three amino acids upstream of the second DRBM. Ser97 was, however, not formally shown to be phosphorylated in our experiments, possibly because it occurs in a long peptide carrying several potential phosphorylation sites (see Fig. 1a). We show that phosphomimetic mutations of PKR Ser6 and Ser97 inhibit PKR activity. Conversely phosphoinhibiting mutations of these residues consistently lead to PKR activation.

It has been proposed that PKR Thr451 phosphorylation by cellular kinases would serve to prime PKR for faster activation upon dsRNA binding^43^. Given the absence of Ser6 phosphorylation observed in cells expressing kinase-dead PKR (Fig. 1a), Ser6 is likely not the target of cellular kinases but rather represents an autophosphorylation site, which would act, like Ser33^41^, as a negative feed-back mechanism. Early autophosphorylation of these residues might counterbalance sensitization by Thr451 phosphorylation and act to dampen PKR activation by endogenous levels of dsRNA or by transient stresses, thus limiting the risk of PKR-mediated autoimmunity exacerbation. We observed the occurrence of PKR Ser6 phosphorylation after infection with a TMEV leader protein mutant known to trigger a strong PKR activation^22^. Since Ser6 appears to be a PKR autophosphorylation site, this residue will likely be phosphorylated in response to other PKR activating stimuli. This, however, remains to be confirmed.

The gnomAD database (v2.1.1)^44^ does not document any variation in Ser97 and only shows the occurrence of synonymous codon variants for Ser6. The fact that only synonymous mutations were identified could support the view that the loss of a phosphorylatable residue might lead to a lethal PKR activation. However, opposite interpretations of the lack of missense mutations of Ser6 and Ser97 could also be formulated.

PACT and the murine Rax homolog were reported to enhance PKR activity in vitro, in the absence of dsRNA^16,17^. Interestingly, protein–protein interaction between PACT and PKR occurs through interaction of the DRBMs^45^. TRBP is another dsRNA binding protein, which can interact with PACT and PKR through its DRBMs^46^. TRBP can inhibit PKR by sequestering dsRNA or PACT as well as through direct protein–protein interaction with PKR^45^. Thus, a structural modification of PKR DRBMs due to Ser6 and Ser97 mutations might have altered the interaction between PKR and PACT or TRBP.

Results presented in Fig. 3 show that PACT is not essential for spontaneous activation of Ser-to-Ala PKR mutants since the S6AS97A mutation increased PKR Thr446 phosphorylation and SG formation, even in PACT-deficient cells. In transfection experiments, PKR is likely overexpressed in some cells, which may limit the amount of endogenous PACT and/or TRBP available for regulation. We were unable to assess PACT interaction with the S6A-S97A PKR mutant in cells transduced with lentiviral vectors that express near-physiological amounts of this mutant because constitutive expression of activated PKR turned out to be toxic for transduced cells. We were however able to immunoprecipitate the S6D-S97D PKR mutant but attempts to detect an interaction between PACT or TRBP and this PKR mutant were unsuccessful, probably due to the anti-PKR antibody directed to the N-terminal region of PKR which might interfere with PKR:PACT or PKR:TRBP interactions. Our data therefore do not rule out the involvement of PACT and of TRBP in the regulation of PKR mutants in more physiological conditions. They however suggest that PACT is not essential for the overactivation observed with the S6A-S97A mutant.

wt and mutant PKR activation in transfected cells may be triggered by endogenous dsRNA that was shown to be detectable in HeLa cells^47^. The involvement of other PKR modulators, such as nc886 was not analyzed in this study.

Given that phosphomimetic mutations inhibit PKR activity, our working model was that the negative charge added to serine 6 and 97 residues by phosphorylation or by phosphomimetic mutations would either prevent RNA binding and consequent PKR activation or tighten the interaction between the dsRNA binding domain and the catalytic domain, thus maintaining PKR in a close conformation, even upon dsRNA binding. This second hypothesis is more likely because the double Ser6- and Ser97-to-Asp mutant, which almost completely lost activity, conserved some ability to bind dsRNA and to dimerize (Figs. 6e, 7c, 8c).

Reciprocally, Ser-to-Ala, phosphoinhibitory mutations increased spontaneous PKR activity, suggesting that these mutations may lower the affinity of the DRBMs for the catalytic domain and thereby facilitate PKR opening and activation (Fig. 8b).

Although several studies pointed to the involvement of DRBM2 only in the interaction with the kinase domain of the protein, our data, in agreement with others^42^, would suggest that both DRBMs contribute to PKR activity inhibition. It is noteworthy that our data were obtained in living cells and, although more difficult to interpret than those obtained in vitro, might sometimes better fit physiological conditions. Further work is clearly required to decipher the complex picture of PKR regulation by phosphorylation, whether by autophosphorylation or by cellular kinase-mediated phosphorylation. This work contributed with the identification of Ser6 and Ser97 as potential target sites allowing either positive or negative PKR activity regulation for therapeutic purposes.

## Methods

### Cell culture

HeLa cells used in this study derive from a HeLa M subclone that reportedly has low endogenous RNase L activity (kindly provided by R. Silvermann)^48^. 293T cells^49^ were a kind gift from F. Tangy. HeLa M and 293T cells were maintained in Dulbecco modified Eagle medium (Lonza) supplemented with 10% fetal bovine serum (FBS) (Sigma), 100 U/ml penicillin and 100 ug/ml streptomycin (Lonza). BHK-21 cells (ATCC) were maintained in Glasgow’s modified Eagle’s medium (GMEM) (Gibco) supplemented with 10% Newborn calf serum (Gibco), 100U penicillin and 100 ug/ml streptomycin, and 2.6 g/L Tryptose Phosphate Broth (Difco). All cells were maintained at 37 °C in 5% CO_2_.

### PKR and PACT gene inactivation

HeLa PKR knock-out cells (PKR-KO) and HeLa PACT-PKR double knock-out (DKO) cells were generated by CRISPR-Cas9 genome editing, using the off-target limiting Cas9 D10A nickase mutant as previously described^50^. Single guide RNAs were identified using the now-defunct MIT design tool (http://crispr.mit.edu) and cloned at the *Bpi*I site of pX461 as hybridized oligonucleotides. Primers targeting exon 1 of human PKR were: sgRNA1 (sense: 5′-CAC CGT ACT CCC TGC TTC TGA-3′, antisense 5′-AAA CTC AGA AGC AGG GAG TAG TAC-3′) and sgRNA2 (sense 5′-CAC CGA TTC AGG ACC TCC ACA TGA T-3′, antisense 5′-AAA CAT CAT GTG GAG GTC CTG AAT C-3′). Primers targeting exon 4 of human PACT were: sgRNA1 (sense 5′-CAC CGT TGG AAG GGT CAG GCA TTA A-3′, antisense 5′-AAA CTT AAT GCC TGA CCC TTC CAA C-3′) and sgRNA2 (sense 5′-CAC CGA AGA ACC AGC TTA ATC CTA T-3′, antisense 5′-AAACATAGGATTAAGCTGGTTCTTC-3′). No off-target was predicted by the MIT sgRNA design tool. For each gene, the pair of pX461 derivatives expressing sgRNA1 and 2 were co-transfected in HeLa cells. Forty-eight hours after transfection, GFP-positive cells were sorted and cloned at a density of 1 cell per well in 96-well plates. Abrogation of PKR or PACT expression was verified by western blotting (see “empty” Fig. 3a).

### Viruses, cell infection and determination of viral titres

The virus used in this study is the KJ6 derivative of Theiler’s murine encephalomyelitis virus (TMEV) persistent strain DA1. It contains capsid mutations that adapt the virus to infect L929 cells more efficiently^51^. FB09 is a KJ6 derivative carrying the M60V mutation in the leader (L) protein, that abrogates PKR antagonism^52^. Viral stocks were produced, as described previously^53^, by transfection of in vitro transcribed RNA derived from the corresponding plasmids (pKJ6 and pFB09). Virus stocks were maintained at − 80 °C. For infection, the virus was diluted in serum-free medium and used at a multiplicity of infection (MOI) of 5 PFU/cell. Cells were washed once with serum-free DMEM and the viral inoculum was overlaid for 1 h at 37 °C. The virus was then removed and cells were washed twice with PBS (Lonza). Complete medium was replenished for 8 or 12 h as specified.

Virus titers were determined by standard plaque assay on BHK-21 cells. Supernatants, collected from FB09 infected cells at 12 hpi, were diluted in serum-free GMEM and inoculated on BHK-21 monolayers for 1 h at 37 °C. Cells were then overlaid with a 1:1 mix of 1% agarose (Seakem LE) melted in H_2_O and 2 × EMEM medium (Lonza) containing 1% fetal bovine serum, 100 U/ml penicillin and 100 µg/ml streptomycin. After 72 h incubation at 37 °C, cells were fixed with 5% formalin and plaques, revealed with crystal violet solution (Merck), were counted.

### Vectors and cell transduction

CRISPR-Cas9, lentiviral and plasmid-based expression vectors used in this study are listed and described in Table 1. HeLa cell lines constitutively expressing PKR variants were generated by lentiviral transduction of PKR knockout cells. To this end, HeLa PKR-KO cells were seeded in 24-well plates at a density of 40,000 cells per well and transduced by three successive rounds of infection with 100 μl of the filtered lentivirus stock. Transduced cells were selected on the basis of their resistance to G418 (Roche) at a concentration of 2 mg/ml and PKR expression was verified by western blotting.

**Table 1:** Plasmids and viruses used in this study.

Name	Parental	Characteristics
**Viruses (TMEV derivatives)**		
KJ6	TMEV DA1	L^WT^; L929 cell-adapted capsid
FB09	TMEV KJ6	L^M60V^; L929 cell-adapted capsid
**Lentiviral and expression vectors 1**		
pTM945	pCCLsin	Prom_CMV_-Empty-IRES-mCherry
pTM952	pCCLsin	Prom_CMV_-Empty-IRES-neo
pYH05	pTM945	Prom_CMV_-huPKR-IRES-mCherry
pYH12	pTM952	Prom_CMV_-huPKR-IRES-neo
pYH31	pTM945	Prom_CMV_-huPKR(S6A)-IRES-mCherry
pYH32	pTM945	Prom_CMV_-huPKR(S6D)-IRES-mCherry
pYH35	pTM952	Prom_CMV_-huPKR(S6D)-IRES-neo
pTC20	pTM945	Prom_CMV_-huPKR(S6AS97A)-IRES-mCherry
pTC21	pTM945	Prom_CMV_-huPKR(S6DS97D)-IRES-mCherry
pTC22	pTM945	Prom_CMV_-huPKR(S6AS97A4KA)-IRES-mCherry
pTC23	pTM945	Prom_CMV_-huPKR(4KA)-IRES-mCherry
pTC24	pTM952	Prom_CMV_-huPKR(S97D)-IRES-neo
pTC25	pTM952	Prom_CMV_-huPKR(S6DS97D)-IRES-neo
pTC27	pTM952	Prom_CMV_-huPKR(K296R)-IRES-neo
pSO1	pTM945	Prom_CMV_-huPKR(S97A)-IRES-mCherry
pSO2	pTM945	Prom_CMV_-huPKR(S97D)-IRES-mCherry
pFW29	pTM952	Prom_CMV_-huPKR_SmBit-IRES-neo
pFW33	pTM952	Prom_CMV_-huPKR_LgBit-IRES-neo
pFW39	pTM952	Prom_CMV_-huPKR(S6AS97A)SmBit-IRES-neo
pFW40	pTM952	Prom_CMV_-huPKR(S6DS97D)SmBit-IRES-neo
pFW41	pTM952	Prom_CMV_-huPKR(4KA)SmBit-IRES-neo
pFW42	pTM952	Prom_CMV_-huPKR(S6AS97A)LgBit-IRES-neo
pFW43	pTM952	Prom_CMV_-huPKR(S6DS97D)LgBit-IRES-neo
pFW44	pTM952	Prom_CMV_-huPKR(4KA)LgBit-IRES-neo
**CRISPR-Cas9 vectors**		
pX461		pSpCas9n(BB)-T2A-GFP
pYH1	pX461	sgRNA1(exon1huPKR)
pYH2	pX461	sgRNA2(exon1huPKR)
pFW19	pX461	sgRNA1(exon4huPACT)
pFW20	pX461	sgRNA2(exon4huPACT)

These plasmids were generated in a lentiviral vector backbone. They are named pTM, pYH, pTC… when used as expression plasmids, and TM, YH, TC… when used as lentiviral vectors.

### Flow cytometry

For cell sorting, cells were dissociated with Trypsin–EDTA (Gibco), washed twice with PBS, resuspended in PBS containing 1% FBS and 1 mM EDTA and kept on ice until sorting. GFP^+^ cells were sorted and cloned at a density of 1 cell per well in 96-well plate using a FACSAria III Cell Sorter (BD Biosciences).

### Plasmid DNA and poly(I:C) transfections

HeLa cell transfection was performed using TransIT-LT1 reagent (Mirus Bio). DNA and transfection reagent were diluted in serum-free medium (Opti-MEM, Gibco). Cells seeded for 24 h were transfected in either 24-well plates using 0.5 μg of DNA and 1.5 μl of transfection reagent per well or in 6 well-plates using 2.5 μg of DNA and 7.5 μl of transfection reagent per well. Cells were harvested 48 h after transfection for immunoprecipitation and/or western blotting analysis. For lentivirus production, 293T cells were seeded the day before in 6 well-plates and transfected with 2.5 μg of a mix of standard lentiviral production vectors using 7.5 μl of TransIT-LT1 reagent (Mirus Bio). Supernatants were harvested 24 h and 48 h after transfection, filtered and maintained at -80 °C until use. For poly(I:C) treatment, cells transfected for 40 h with PKR mutants expressing plasmids were re-transfected with 2 μl of poly(I:C) (stock at 2 mg/ml) using 5 μl Lipofectamine 2000 (Invitrogen). Cells were harvested 8 h after transfection.

### Western blotting

Proteins extracted in 1 × Laemmli buffer were heated at 95 °C for 10 min, run on 8 or 10% Tris–glycine sodium dodecyl sulfate polyacrylamide gels and transferred onto polyvinylidene fluoride membranes (Immobilon-P, Millipore). Membranes were blocked in Tris-buffered saline (TBS) containing 5% non-fat dry milk (TBS-milk) for 1 h at room temperature. Blots were probed with primary antibodies diluted in TBS-milk overnight at 4 °C and then washed three times with TBS containing 0.1% Tween-20 (TBST) for 15 min at room temperature. Anti-PKR (rabbit, 18244-1-AP, Proteintech) 1:4000, anti-phospho T446 PKR (rabbit, ab32036, Abcam) 1:4000, anti-phospho PKR T451 (rabbit, 81,303, Abcam) 1:2000, anti-ADAR1 (rabbit, 14,175, Cell Signalling Technology) 1:3000, anti-eIF2α (rabbit, 9722 Cell signalling Technology) 1:1000, anti-phospho eIF2α Ser51 (rabbit, 3398 Cell Signalling Technology) 1:1000, anti-cleaved PARP Asp214 (rabbit, 5625 Cell Signalling Technology) 1:1000, anti-β actin (mouse, 5441, Sigma) 1:10,000, anti-TMEV 3D polymerase (rabbit, kindly provided by M. Brahic) 1:1000, anti-PACT (mouse, sc-377103, Santa Cruz Biotechnology) 1:200. Primary antibodies were detected using goat anti-rabbit or goat anti-mouse IgG (H + L) HRP-conjugated antibodies (1:2000, Dako). After one hour of incubation at room temperature, blots were washed three times for 15 min with TBST and revealed with SuperSignal West Chemiluminescent Substrate (Pico or Dura, Thermo Scientific). Blot luminescence was acquired with a cooled CCD camera (Fusion Solo-S, Vilber) and quantified within the limits of the dynamic range, using software Bio1D version 15.08.

### Immunostaining

Transfected or infected cells (as specified in figure legends) seeded in 96 wells-plates were fixed with PBS containing 4% paraformaldehyde for 5 min at room temperature. Cells were then washed with PBS and permeabilized with PBS-0.1% Triton X-100 (ICN Biomedicals Inc.) for 5 min at room temperature. Cells were then blocked with TNB blocking reagent (Perkin Elmer) for 1 h at room temperature. Primary antibodies, diluted in TNB reagent, were incubated for 1 h at room temperature at the following dilutions: anti-PKR (rabbit, 18244-1-AP, Proteintech) 1:400, anti-eif3η (mouse, sc-137214 Santa Cruz) 1:800. Cells were then washed three times for 5 min in PBS-0.1% Tween 20 and incubated with species-matched secondary antibodies (Alexa Fluor 488- and 697-conjugated antibodies, Molecular Probes) 1:800 for 1 h at room temperature. Cells were washed three times for 5 min in PBS-0.2% Tween 20 and maintained in PBS-azide 0.02% until analysis. Fluorescence analysis was performed with a spinning disk confocal microscope (Zeiss, Germany). Image acquisition and processing (intensity, contrast and pseudocolours) were done with the Zen image acquisition sofware (Zeiss, Germany) using the same parameters across micrographs.

### PKR immunoprecipitation

HeLa cells and PKR-KO cells transduced with the lentiviral vector TC27 expressing a kinase-dead PKR mutant (K296R) were seeded in 10 cm diameter culture dishes. Both cell lines were either mock-infected or infected for 10 h with 5 PFU per cell of virus FB09. After infection, cells were washed three times with cold PBS and lysed for 15 min on ice with 800 μl of lysis buffer (150 mM NaCl, 50 mM Tris pH 7.5, 1 mM EDTA, 1% NP40, 1 mM PMSF) containing 1 tablet of phosphatase/protease inhibitor cocktail (Pierce—Thermo Scientific) per 10 ml of lysis buffer. Cell lysates were homogenized by 10 passages through 21G needles and cleared by centrifugation at 12,000 × *g* for 10 min at 4 °C. Supernatants were then transferred to new 1.5 ml tubes and non-specific binding was removed by incubating supernatants with 20 μl of protein A/G magnetic beads (Pierce) for 1 h at 4 °C. Cleared supernatants were collected by centrifugation and a 20 μl aliquot per condition was mixed with 10 μl of 3 × Laemmli buffer as a “total cell lysate” control. Remaining supernatants were then incubated with 20 μl of anti-PKR magnetic beads (Cell Signalling Technology) for 2 h at 4 °C. Beads were washed three times for 5 min at 4 °C with 500 μl of lysis buffer and resuspended in 30 μl of 1.5 × Laemmli buffer.

### Poly(I:C) pull down assay

Cells seeded in 6 well-plates and transfected for 48 h with PKR expressing plasmids were lysed with 150 μl of lysis buffer (same as above, with the addition of RNase Inhibitor 1:1600, Thermo Scientific) for 15 min at 4 °C. Cells lysates were homogenized by repeated passages through 21G needles and cleared by centrifugation at 12,000 × *g* for 10 min at 4 °C. A sample of 10 μl was mixed with 5 μl of 3 × Laemmli buffer as input control. Half sample per condition was incubated with 5 μl of Biotinylated poly(I:C) (poly(I:C) HMW BIOTIN, InvivoGen) (stock at 10 mg/ml) or 5 μl water for 1 h at 30 °C. The samples were then incubated with 10 μl of anti-Streptavidin magnetic beads (Pierce) for 30 min at 4 °C and then washed three times for 5 min at 4 °C with lysis buffer. Beads were resuspended in 30 μl 1.5 × Laemmli buffer and analysed by western blotting.

### Nanoluciferase (dimerization) assay

HeLa PKR-KO cells were seeded in 24-well plates at a density of 25,000 cells/well and co-transfected with 0.25 μg of plasmid expressing PKR variants fused to the small subunit (SmBit) of nanoluciferase and 0.25 μg of plasmid expressing PKR variants fused to the large subunit of nanoluciferase (LgBit), using 1.5 μl of TransIT-LT1 reagent. After 22 h cells were re-transfected with 2 μg of poly(I:C) using 5 μl Lipofectamine 2000. Cells were lysed 2 h after transfection. Luciferase assays were performed according to the manufacturer’s recommendations (Promega N1130) and luminescence was measured with a Glomax 20/20 Luminometer (Promega), 5′ and 20′ after the addition of the lysis buffer/substrate mix.

### Mass spectrometry

Mass spectometry analysis was performed as described previously^54,55^. After immunoprecipitation, samples were resolved using a 10% Tris–Glycine SDS-PAGE and proteins were visualized using PageBlue (Thermo Scientific, 24,620). Bands of interest were cut out from the gel and digested with trypsin (50 ng/μl in 50 mM NH4HCO3 buffer, pH 8.0). The peptides were analysed by capillary LC-tandem mass spectrometry using an Orbitrap Fusion Lumos ion trap mass spectrometer (ThermoScientific, San Jose, CA) fitted with a nanoelectrospray probe. The data were analysed with the ProteomeDiscoverer software (ThermoScientific, version 2.4), and the proteins were identified with SequestHT against target-decoy non-redundant human protein database obtained from Uniprot. The following parameters were used: trypsin was selected with proteolytic cleavage only after arginine and lysine, number of internal cleavage sites was set to 1, mass tolerance for precursors was 10 ppm and 0.5 Da for fragment ions, considered dynamic modifications were + 15.994 Da for oxidized methionine and + 79.966 for phosphorylated Ser, Thr and Tyr. Peptide matches were filtered using the q-value and Posterior Error Probability calculated by the Percolator algorithm ensuring an estimated false positive rate below 5%. The filtered Sequest HT output files for each peptide were grouped according to the protein from which they were derived. The mass spectrometry proteomics data have been deposited to the ProteomeXchange Consortium via the PRIDE^56^ partner repository with the dataset identifier PXD022564 and 106019/PXD022564.

### Statistical analysis

Statistical analysis was performed using Prism 7 (GraphPad Software). Error bars represent the standard error of the mean. One-way ANOVA with Dunnett’s post hoc tests or two-way ANOVA with Sidak’s post hoc tests were used as indicated. Statistical significance was considered if *p* value was inferior to 0.05. *p* values: ****p ≤ 0.0001, ***p ≤ 0.001, **p ≤ 0.01, *p ≤ 0.05, ns = not significant.

## Supplementary Information

Supplementary Figures.

## References

[1] Farrell PJ, Balkow K, Hunt T, Jackson RJ, Trachsel H (1977). Phosphorylation of initiation factor elF-2 and the control of reticulocyte protein synthesis. Cell.

[2] Anderson P, Kedersha N (2009). Stress granules. Curr. Biol..

[3] Garcia MA, Meurs EF, Esteban M (2007). The dsRNA protein kinase PKR: Virus and cell control. Biochimie.

[4] Meurs E (1990). Molecular cloning and characterization of the human double-stranded RNA-activated protein kinase induced by interferon. Cell.

[5] Thomis DC, Doohan JP, Samuel CE (1992). Mechanism of interferon action: cDNA structure, expression, and regulation of the interferon-induced, RNA-dependent P1/eIF-2 alpha protein kinase from human cells. Virology.

[6] Nanduri S, Rahman F, Williams BR, Qin J (2000). A dynamically tuned double-stranded RNA binding mechanism for the activation of antiviral kinase PKR. EMBO J..

[7] Robertson HD, Mathews MB (1996). The regulation of the protein kinase PKR by RNA. Biochimie.

[8] Nanduri S, Carpick BW, Yang Y, Williams BR, Qin J (1998). Structure of the double-stranded RNA-binding domain of the protein kinase PKR reveals the molecular basis of its dsRNA-mediated activation. EMBO J..

[9] Bevilacqua PC, Cech TR (1996). Minor-groove recognition of double-stranded RNA by the double-stranded RNA-binding domain from the RNA-activated protein kinase PKR. Biochemistry.

[10] McMillan NA (1995). Mutational analysis of the double-stranded RNA (dsRNA) binding domain of the dsRNA-activated protein kinase, PKR. J. Biol. Chem..

[11] Wu S, Kaufman RJ (1997). A model for the double-stranded RNA (dsRNA)-dependent dimerization and activation of the dsRNA-activated protein kinase PKR. J. Biol. Chem..

[12] Dey M (2005). Mechanistic link between PKR dimerization, autophosphorylation, and eIF2alpha substrate recognition. Cell.

[13] Romano PR, Green SR, Barber GN, Mathews MB, Hinnebusch AG (1995). Structural requirements for double-stranded RNA binding, dimerization, and activation of the human eIF-2 alpha kinase DAI in *Saccharomyces cerevisiae*. Mol. Cell. Biol..

[14] Romano PR (1998). Inhibition of double-stranded RNA-dependent protein kinase PKR by vaccinia virus E3: Role of complex formation and the E3 N-terminal domain. Mol. Cell. Biol..

[15] Dey M, Mann BR, Anshu A, Mannan MA (2014). Activation of protein kinase PKR requires dimerization-induced cis-phosphorylation within the activation loop. J. Biol. Chem..

[16] Ito T, Yang M, May WS (1999). RAX, a cellular activator for double-stranded RNA-dependent protein kinase during stress signaling. J. Biol. Chem..

[17] Patel RC, Sen GC (1998). PACT, a protein activator of the interferon-induced protein kinase, PKR. EMBO J..

[18] Dabo S (2017). Inhibition of the inflammatory response to stress by targeting interaction between PKR and its cellular activator PACT. Sci. Rep..

[19] Bennett RL (2006). RAX, the PKR activator, sensitizes cells to inflammatory cytokines, serum withdrawal, chemotherapy, and viral infection. Blood.

[20] Gale M, Tan SL, Wambach M, Katze MG (1996). Interaction of the interferon-induced PKR protein kinase with inhibitory proteins P58IPK and vaccinia virus K3L is mediated by unique domains: Implications for kinase regulation. Mol. Cell. Biol..

[21] Park H (1994). TAR RNA-binding protein is an inhibitor of the interferon-induced protein kinase PKR. Proc. Natl. Acad. Sci. USA.

[22] Borghese F, Sorgeloos F, Cesaro T, Michiels T (2019). The leader protein of Theiler's virus prevents the activation of PKR by dsRNA. J. Virol..

[23] Liu CX (2019). Structure and degradation of circular RNAs regulate PKR activation in innate immunity. Cell.

[24] Schlee M, Hartmann G (2016). Discriminating self from non-self in nucleic acid sensing. Nat. Rev. Immunol..

[25] Wang WJ, Yin SJ, Rong RQ (2015). PKR and HMGB1 expression and function in rheumatoid arthritis. Genet. Mol. Res..

[26] Rigbolt KT (2011). System-wide temporal characterization of the proteome and phosphoproteome of human embryonic stem cell differentiation. Sci. Signal..

[27] Katze MG (1991). Functional expression and RNA binding analysis of the interferon-induced, double-stranded RNA-activated, 68,000-Mr protein kinase in a cell-free system. Mol. Cell. Biol..

[28] Marques JT, White CL, Peters GA, Williams BR, Sen GC (2008). The role of PACT in mediating gene induction, PKR activation, and apoptosis in response to diverse stimuli. J. Interferon Cytokine Res..

[29] Lee SB, Esteban M (1994). The interferon-induced double-stranded RNA-activated protein kinase induces apoptosis. Virology.

[30] Dixon AS (2016). NanoLuc complementation reporter optimized for accurate measurement of protein interactions in cells. ACS Chem. Biol..

[31] Balachandran S (2000). Essential role for the dsRNA-dependent protein kinase PKR in innate immunity to viral infection. Immunity.

[32] Stojdl DF (2000). The murine double-stranded RNA-dependent protein kinase PKR is required for resistance to vesicular stomatitis virus. J. Virol..

[33] Crow YJ, Manel N (2015). Aicardi-Goutieres syndrome and the type I interferonopathies. Nat. Rev. Immunol..

[34] Chung H (2018). Human ADAR1 prevents endogenous RNA from triggering translational shutdown. Cell.

[35] Garcia-Ortega MB (2017). Clinical and therapeutic potential of protein kinase PKR in cancer and metabolism. Expert Rev. Mol. Med..

[36] Lee YS, Kunkeaw N, Lee YS (2020). Protein kinase R and its cellular regulators in cancer: An active player or a surveillant?. Wiley Interdiscip. Rev. RNA.

[37] Freundt EC, Drappier M, Michiels T (2018). Innate immune detection of cardioviruses and viral disruption of interferon signaling. Front. Microbiol..

[38] Maarifi G, El Asmi F, Maroui MA, Dianoux L, Chelbi-Alix MK (2018). Differential effects of SUMO1 and SUMO3 on PKR activation and stability. Sci. Rep..

[39] Calderon BM, Conn GL (2017). Human noncoding RNA 886 (nc886) adopts two structurally distinct conformers that are functionally opposing regulators of PKR. RNA.

[40] Lee K (2011). Precursor miR-886, a novel noncoding RNA repressed in cancer, associates with PKR and modulates its activity. RNA.

[41] Taylor DR (2001). Hepatitis C virus envelope protein E2 does not inhibit PKR by simple competition with autophosphorylation sites in the RNA-binding domain. J. Virol..

[42] Wang D (2017). Auto-phosphorylation represses protein kinase R activity. Sci. Rep..

[43] Zykova TA, Zhu F, Zhang Y, Bode AM, Dong Z (2007). Involvement of ERKs, RSK2 and PKR in UVA-induced signal transduction toward phosphorylation of eIF2alpha (Ser(51)). Carcinogenesis.

[44] Karczewski KJ (2020). The mutational constraint spectrum quantified from variation in 141,456 humans. Nature.

[45] Singh M, Castillo D, Patel CV, Patel RC (2011). Stress-induced phosphorylation of PACT reduces its interaction with TRBP and leads to PKR activation. Biochemistry.

[46] Laraki G (2008). Interactions between the double-stranded RNA-binding proteins TRBP and PACT define the Medipal domain that mediates protein-protein interactions. RNA Biol..

[47] Dhir A (2018). Mitochondrial double-stranded RNA triggers antiviral signalling in humans. Nature.

[48] Dong B, Niwa M, Walter P, Silverman RH (2001). Basis for regulated RNA cleavage by functional analysis of RNase L and Ire1p. RNA.

[49] DuBridge RB (1987). Analysis of mutation in human cells by using an Epstein–Barr virus shuttle system. Mol. Cell. Biol..

[50] Jacobs S (2018). Species specificity of type III interferon activity and development of a sensitive luciferase-based bioassay for quantitation of mouse interferon-lambda. J. Interferon Cytokine Res..

[51] Jnaoui K, Michiels T (1998). Adaptation of Theiler's virus to L929 cells: Mutations in the putative receptor binding site on the capsid map to neutralization sites and modulate viral persistence. Virology.

[52] Ricour C (2009). Random mutagenesis defines a domain of Theiler's virus leader protein that is essential for antagonism of nucleocytoplasmic trafficking and cytokine gene expression. J. Virol..

[53] Michiels T, Dejong V, Rodrigus R, Shaw-Jackson C (1997). Protein 2A is not required for Theiler's virus replication. J. Virol..

[54] Bollaert E (2018). HBP1 phosphorylation by AKT regulates its transcriptional activity and glioblastoma cell proliferation. Cell. Signal..

[55] Tossounian MA (2020). Methionine sulfoxide reductase B from *Corynebacterium diphtheriae*catalyzessulfoxide reduction via an intramolecular disulfide cascade. J. Biol. Chem..

[56] Perez-Riverol Y (2019). The PRIDE database and related tools and resources in 2019: Improving support for quantification data. Nucleic Acids Res..

