# Supplementary material for: PKR activity modulation by phosphomimetic mutations of serine residues located three aminoacids upstream of double-stranded RNA binding motifs
Source: Sci Rep. 2021 Apr 28;11:9188. doi: 10.1038/s41598-021-88610-z (PMC8080564; doi:10.1038/s41598-021-88610-z)
Supplement: Supplementary file 1 — Supplementary Figures. [file 41598_2021_88610_MOESM1_ESM.pdf]

Supplementary information

PKR activity modulation by phosphomimetic mutations of serine residues located three aminoacids upstream of double-stranded RNA binding motifs.

Teresa Cesaro, Yohei Hayashi, Fabian Borghese, Didier Vertommen, Fanny Wavreil and Thomas Michiels.

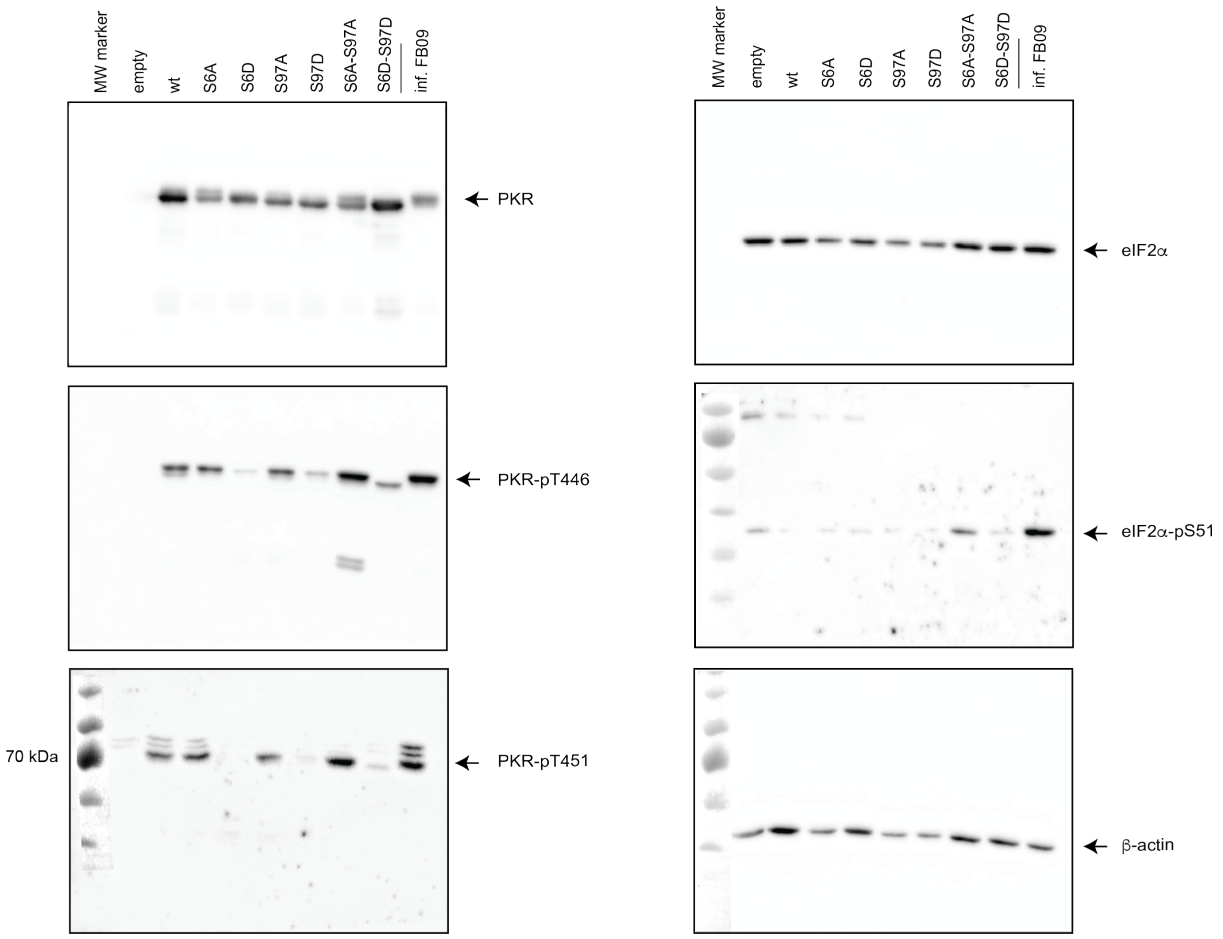

Figure S1. **Mutations of Ser6 and Ser97 regulate PKR activation.**
Full-size images of the western blots in support to Fig. 2a, showing total and phosphorylated forms of PKR and eIF2α in PKR-KO HeLa cells, 48 hours after transfection of plasmids expressing the indicated PKR variants. β-actin was detected as a loading control.

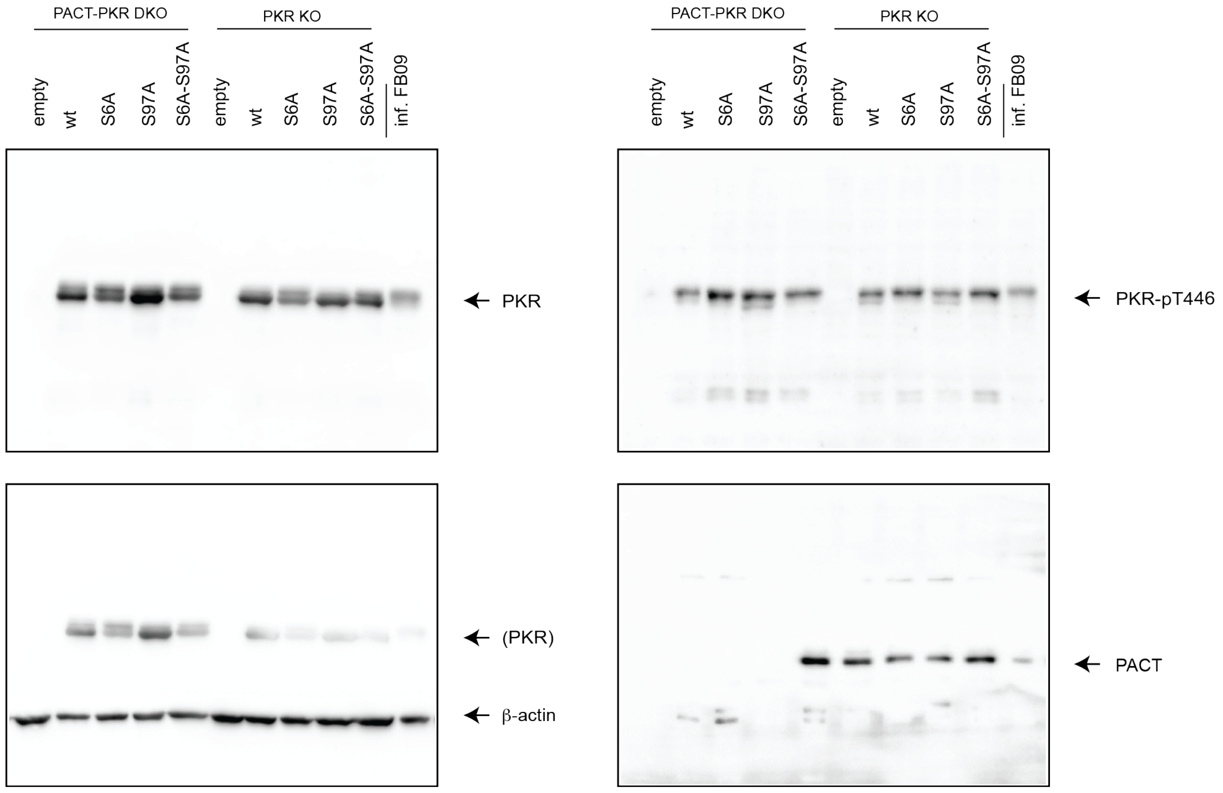

Figure S2. **PACT is not required for spontaneous activation of Ser6- and Ser97-to-Ala PKR mutants.**
Full-size images of the western blots in support to Fig. 3a, showing total and phosphorylated forms of PKR, as well as PACT in PKR-KO HeLa cells, 48 hours after transfection of plasmids expressing the indicated PKR variants. β-actin was detected as a loading control. Note that β-actin (lower left panel) was detected by reprobing the membrane that was used for PKR detection (upper left panel) so that PKR is still visible on the blot.

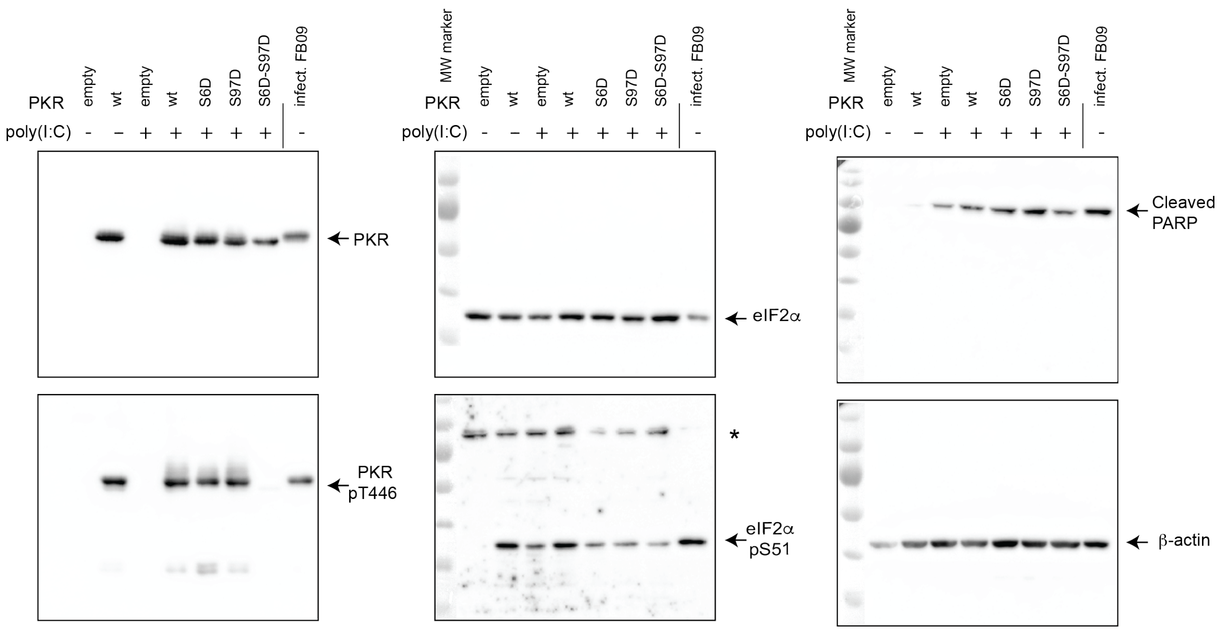

Figure S3. **Combination of S6D and S97D phosphomimetic mutations prevents PKR activation by poly(I:C).**

Full-size images of the western blots in support to Fig. 5a. Total protein samples were isolated from PKR-KO cells transfected for 40h with plasmids expressing the indicated PKR constructs and subsequently transfected for 8h with (+) or without (-) poly(I:C). The unrelated infect.FB09 sample (HeLa cells infected with L^{M60V}-mutant TMEV for 12h) was used as positive control for phospho-PKR and phospho-eIF2α detection. Left panels: PKR and phospho-Thr446 PKR detection; Central panels: eIF2α and phospho-Ser51 eIF2α detection. The asterisk indicates non-specific detection of a high-molecular weight product, which is dependent on sample preparations as it is less detected in the unrelated sample of FB09-infected cells; Right panels: Cleaved PARP was detected as a marker for apoptosis. β-actin was detected as a loading control.

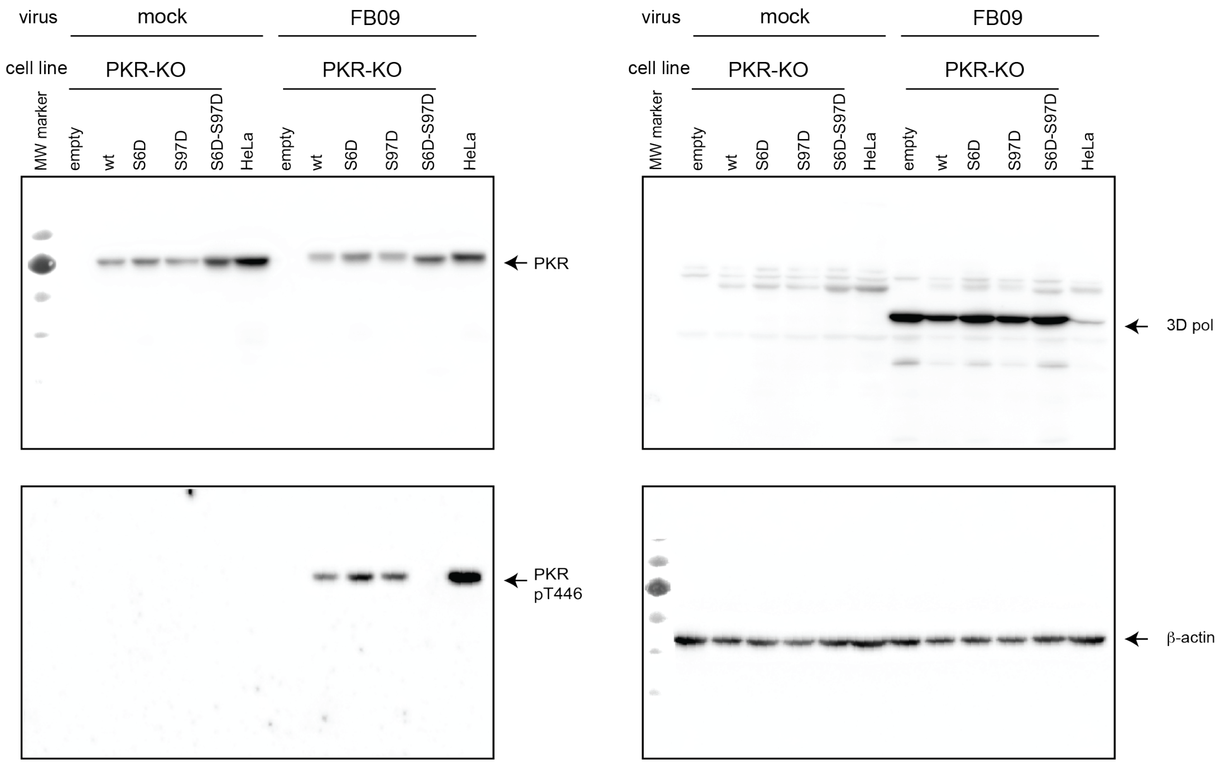

Figure S4. **Combination of S6D and S97D phosphomimetic mutations prevents PKR activation by viral infection.**
Full-size images of the western blots in support to Fig. 6a, showing detection of total and phosphorylated forms of PKR, and of polymerase (3D pol). Samples were from HeLa cells and PKR-KO HeLa cells transduced to stably express the indicated PKR variants and subsequently either mock-infected or infected for 12h with the L^{M60V} TMEV mutant (FB09). β-actin was detected as a loading control.

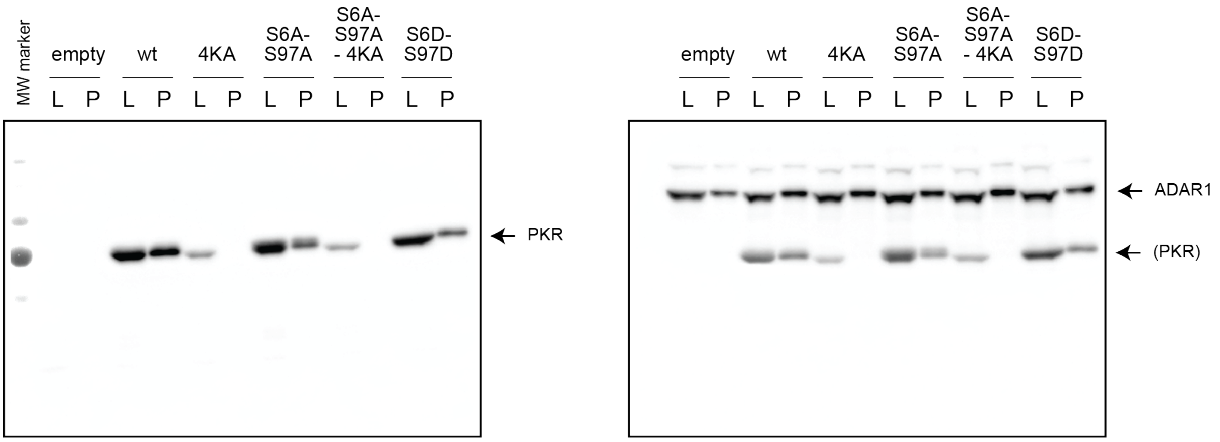

Figure S5. **Ser6 and Ser97 mutations slightly affect dsRNA binding**
Full-size images of the western blots in support to Fig. 6e, showing detection of PKR (left) and ADAR1 (right) in lysates (L) and after poly(I:C) pull down (P). Samples were prepared from PKR-KO cells transfected for 48h with plasmids expressing wt or indicated PKR mutants. Note that ADAR1 detection was obtained by reprobing the membrane used for PKR detection so that PKR is also visible on the blot of ADAR1 (right panel).

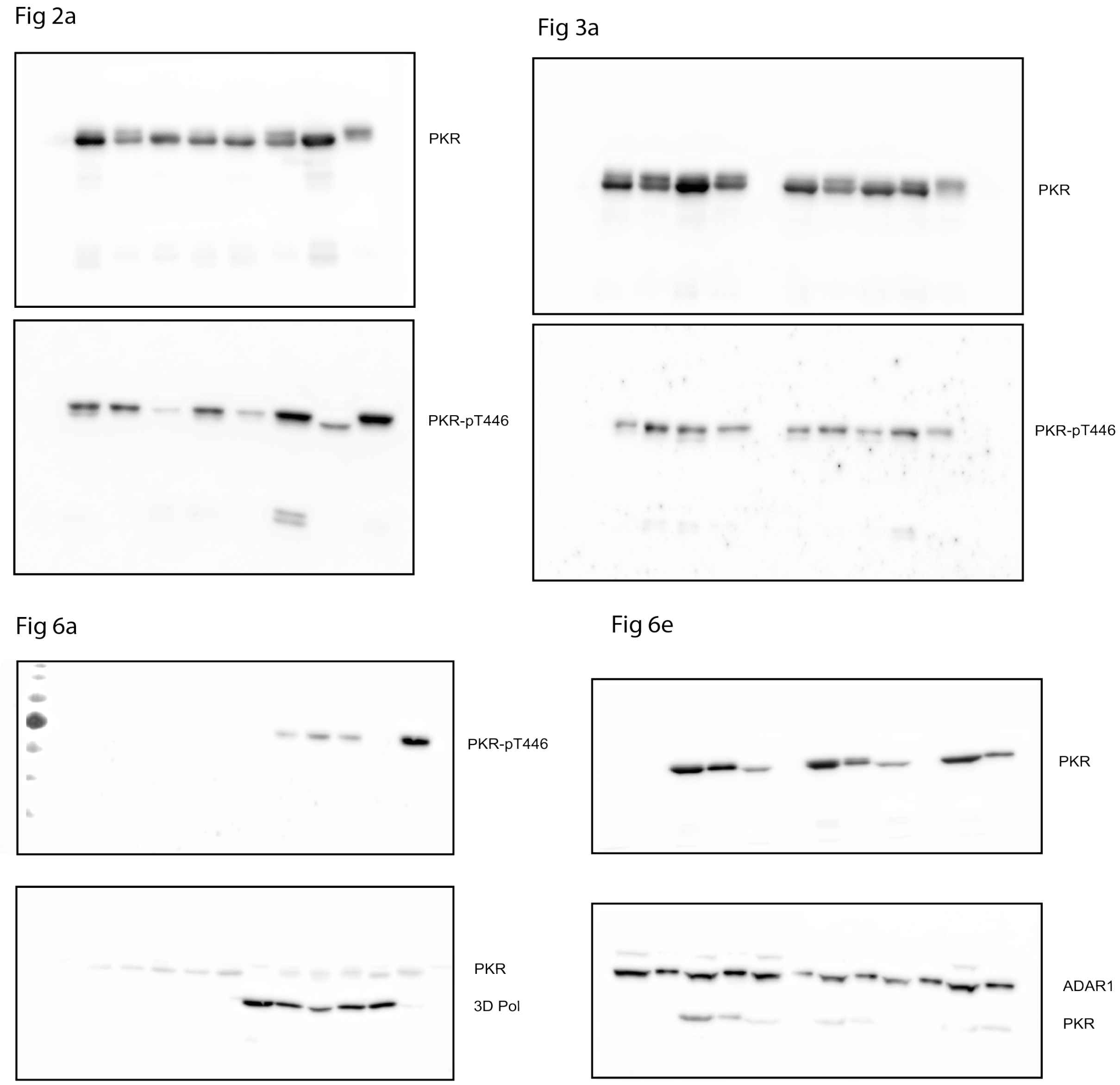

Figure S6. Western blot images acquired with shorter exposure times.
